# Effects of Growth Period and Storage Methods on Primary Metabolite Contents and Antioxidant Activities of *Morus alba* L. Leaf

**DOI:** 10.3390/molecules28010148

**Published:** 2022-12-24

**Authors:** Lei Hu, Dandan Chen, Wei Zhou, Xiaoyang Chen, Qing Zhang

**Affiliations:** 1 Guangdong Key Laboratory for Innovative Development and Utilization of Forest Plant Germplasm, State Key Laboratory for Conservation and Utilization of Subtropical Agro-bioresources, South China Agricultural University, Guangzhou 510642, China; 2 Guangdong Research and Development Center of Modern Agriculture (Woody Forage) Industrial Technology, South China Agricultural University, Guangzhou 510642, China; 3 College of Forestry and Landscape Architecture, Guangdong Province Research Center of Woody Forage Engineering Technology, South China Agricultural University, Guangzhou 510642, China

**Keywords:** white mulberry, metabolism, harvesting period, storage method, anti-oxidation ability

## Abstract

(1) Background: Mulberry leaves have been widely consumed due to their richness in bioactive substances and high antioxidant activity. The choice of storage method to ensure the quality of mulberry leaves is a challenge in the supply process. (2) Methods: The differences in primary metabolites of freeze-dried mulberry leaf powder after 30 days of storage under different storage conditions (i.e., vacuum or non-vacuum, 4 °C or room temperature) were investigated. (3) Results: A low temperature and vacuum had better preservation effects on the types and activity of the primary metabolites of mulberry leaves, with vacuum preservation being the best. However, the types of primary metabolites in mulberry leaves were significantly reduced after non-vacuum storage at room temperature compared to those with other storage methods. Among the metabolites detected, including dehydroascorbic acid, various phenolic acids, amino acids, lipids, and carbohydrates showed a significant decrease in their contents of more than 40%, and there was a significant increase in the contents of various compounds of the muconic acid biosynthetic pathway compared to those in other storage methods. Moreover, the antioxidant activity of mulberry leaves stored at room temperature under non-vacuum conditions was also significantly reduced. (4) Conclusions: Vacuum storage is the most ideal storage method for preserving mulberry leaves.

## 1. Introduction

Mulberry (*Morus alba* L.) is a woody plant of the genus Morus in the family Moraceae [1]. Mulberry leaves serve as important sources of macronutrients, micronutrients, and organic acids for us. They can be eaten as vegetables and are considered more flavorful than other leafy vegetables (e.g., amaranth and spinach) [2]. Phytochemical studies have shown that mulberry leaves are rich in ascorbic acid, minerals, and protein. Moreover, the protein content is substantially greater than that in other leafy green vegetables [3]. Proton nuclear magnetic resonance detection found that the primary metabolic extract of mulberry leaves includes four amino acids (alanine (C_3_H_7_NO_2_), proline (C₅H₉NO_2_), asparagine (C_4_H_8_N_2_O_3_), and γ-aminobutyric acid (C_4_H_9_NO_2_)), three organic acids (acetic acid (CH_3_COOH), succinic acid (C_4_H_6_O_4_), and fumaric acid (C_4_H_4_O_4_)), two carbohydrates (glucose (C_6_H_12_O_6_) and sucrose (C_12_H_22_O_11_)) [4], and eight phenolic acids (caffeic acid (C_9_H_8_O_4_), gallic acid (C_7_H_6_O_5_), protocatechuic acid (C_7_H_6_O_4_), vanillic acid (C_8_H_8_O_4_), chlorogenic acid (C_16_H_18_O_9_), and ferulic acid (C_10_H_10_O_4_)) [5]. Notably, γ-aminobutyric acid is the main component isolated from mulberry leaves, and it plays an important role in the development of the nervous system and brain [4].

The primary metabolites in mulberry leaves are beneficial to human health. Thousands of years ago, mulberry leaves were used as an herbal medicine. Recent medical studies have shown that mulberry leaves are also used as a treatment for diseases such as cancer, hypertension, diabetes, and atherosclerosis [5]; e.g., mulberry leaf extract (Reducose) significantly reduced the increase in plasma glucose in volunteers after sucrose intake over 120 minutes, with a percentage reduction range of 42% to 45% compared to a placebo. Moreover, the total insulin rise was significantly suppressed. None of the participants reported any side effects during the course of the study [6]. However, mulberry leaves are highly perishable once they are harvested, and the content of primary metabolites in them can change with the storage conditions and time. Proper storage maximizes the retention of the nutrients of mulberry leaves. It is well known that there are complex physicochemical reactions that are caused by multiple factors (light, air, moisture, temperature and packaging conditions, etc.) involved in food processing and storage [7]. These reactions can not only alter the taste and flavor of foods, but can also significantly affect their nutritional value [7]. Information about the primary metabolites of mulberry leaves stored in different conditions is limited. Therefore, it is necessary to identify good storage conditions for mulberry leaves after they have been harvested to ensure the biological activity of the primary metabolites. 

## 2. Results

### 2.1. Comparison of the Primary Metabolites of Mulberry Leaf after Storage

**Figure 1:**
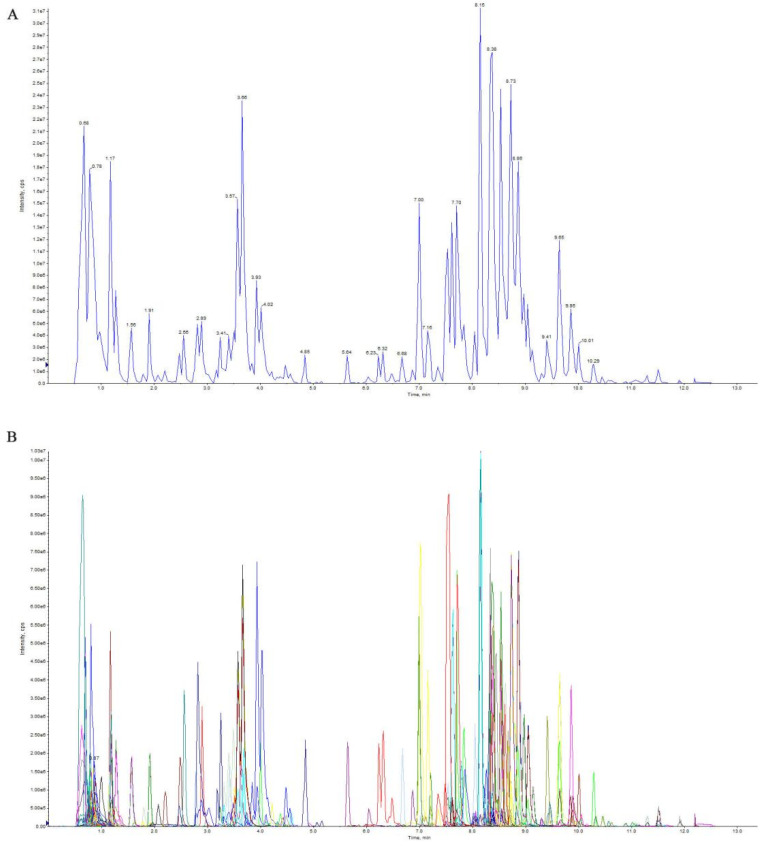
Total ion current of one quality-control sample according to mass spectrometry detection (**A**) and the multi-peak detection plot of metabolites in the multiple-reaction-monitoring mode (**B**).

UPLC-MS/MS was used to detect the types of primary metabolites of mulberry leaves after storage under different conditions. Figure 1 shows the total and extracted ion currents, which indicated that the signal was stable when multiple metabolites were detected in the same sample. The instrument’s stability guaranteed the repeatability and reliability of our metabolomic data.

#### 2.1.1. Qualitative and Quantitative Analyses of Primary Metabolites

**Figure 2:**
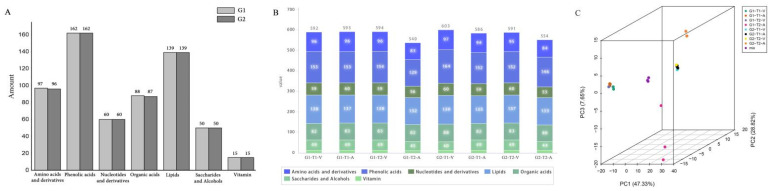
The effects of storage methods on the primary metabolites of G1 and G2. (**A**) The amount of each kind of primary metabolite in G1 and G2. (**B**) Stacked graphs of the amounts of various primary metabolites in the different storage methods of G1 and G2. (**C**) PCA on the effects of storage methods on the primary metabolites of G1 and G2.

In multiple-reaction-monitoring (MRM) mode, UPLC-MS/MS was used to evaluate compounds in each of the four samples in G1 and G2 (eight subgroups). Qualitative analysis indicated that 611 primary metabolites (97 amino acids and derivatives, 162 phenolic acids, 60 nucleotides and derivatives, 88 organic acids, 139 lipids, 50 saccharides and alcohols, and 15 vitamins) were present in G1, while 609 primary metabolites (96 amino acids and derivatives, 162 phenolic acids, 60 nucleotides and derivatives, 87 organic acids, 139 lipids, 50 saccharides and alcohols, and 15 vitamins) were present in G2 (Figure 2A). Among them, G1-T1-V, G1-T1-A, G1-T2-V, and G1-T2-A were determined to exhibit 592, 593, 594, and 540 primary metabolites, respectively (Figure 2B); G2-T1-V, G2-T1-A, G2-T2-V, and G2-T2-A were determined to exhibit 603, 586, 591, and 554 primary metabolites, respectively (Figure 2B). PCA demonstrated that in G1 and G2, T1-V, T1-A, and T2-V formed a cluster and T2-A formed a cluster alone (Figure 2C).

**Figure 3:**
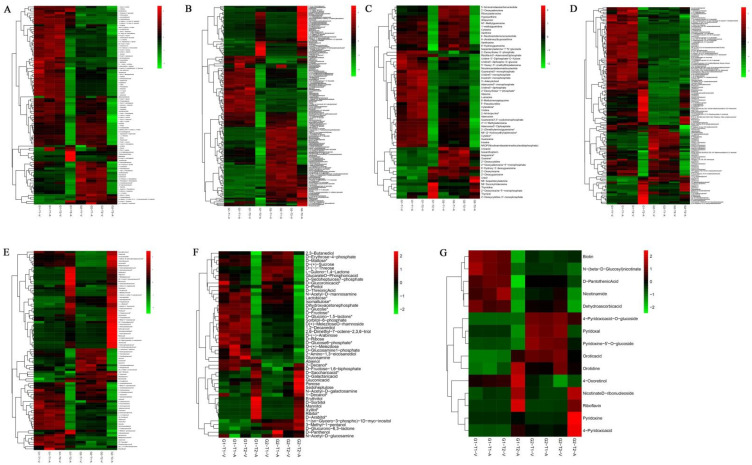
Heatmap of the primary metabolites in the different storage methods of G1 and G2: (**A**) amino acids and derivatives, (**B**) phenolic acids, (**C**) nucleotides and derivatives, (**D**) lipids, (**E**) organic acids, (**F**) saccharides and alcohols, and (**G**) vitamins. *n* = 3.

To confirm the preservation of mulberry leaf quality under various storage conditions, cluster analysis was used to compare the primary metabolites of mulberry leaf according to the storage conditions. Hierarchical clustering analysis revealed that T2-A was clearly distinct from the other groups (Figure 3). The figure shows that vacuum packaging and low-temperature storage were desired for preserving mulberry leaf’s primary metabolites, as they inhibited the loss of primary metabolites and minimized qualitative changes. The primary metabolites in mulberry leaves that were not stored in a vacuum and were at room temperature showed significant differences compared to those in other storage environments, with more than 40% of the assessed primary metabolites being significantly decreased.

#### 2.1.2. Differentially Accumulated Metabolite (DAM) Identification and Functional Annotation Analysis

**Figure 4:**
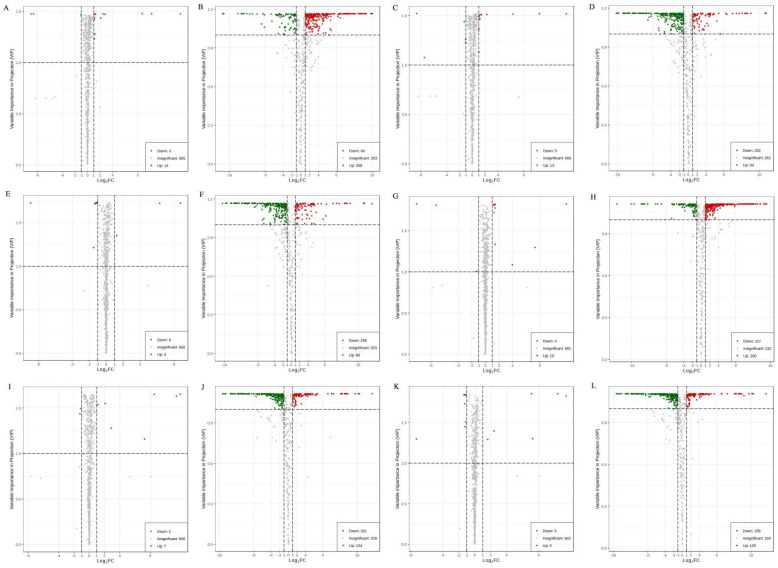
The volcano plots show the content levels of primary metabolites in the different storage methods of G1 and G2. (A–F): in G1. (**A**): T1-V vs. T1-A, (**B**): T2-A vs. T2-V, (**C**): T1-V vs. T2-V, (**D**): T1-A vs. T2-A, (**E**): T1-A vs. T2-V, and (**F**): T1-V vs. T2-A. (**G**–**L**): in G2. (**G**): T1-V vs. T1-A, (**H**): T2-A vs. T2-V, (**I**): T1-V vs. T2-V, (**J**): T1-A vs. T2-A, (**K**): T1-A vs. T2-V, and (**L**): T1-V vs. T2-A.

To determine the differences in the primary metabolites of mulberry leaf according to the storage conditions, the DAMs of G1 and G2 were analyzed with variable importance in projection (VIP) ≥ 1 and a fold change of ≥2 or ≤0.5, and volcano plots were generated (Figure 4).

In G1 (Figure 4A–F), for T1-V versus T1-A, 14 and 3 of the 602 metabolites were increased (2.32%) and decreased (0.50%), respectively. For T2-A versus T2-V, 259 and 88 of the 610 metabolites were increased (42.46%) and decreased (14.43%), respectively. For T1-V versus T2-V, 13 and 5 of the 603 metabolites were increased (2.16%) and decreased (0.83%), respectively. For T1-A versus T2-A, 84 and 262 of the 608 metabolites were increased (13.82%) and decreased (43.09%), respectively. For T1-A versus T2-V, 3 and 8 of the 599 metabolites were increased (0.50%) and decreased (1.34%), respectively. For T1-V versus T2-A, 98 and 259 of the 610 metabolites were increased (16.07%) and decreased (42.46%), respectively.

In G2 (Figure 4G–L), for T1-V versus T1-A, 10 and 3 of the 594 metabolites were increased (1.68%) and decreased (0.51%), respectively. For T2-A versus T2-V, 260 and 117 of the 609 metabolites were increased (42.69%) and decreased (19.21%), respectively. For T1-V versus T2-V, 7 and 2 of the 597 metabolites were increased (1.17%) and decreased (0.34%), respectively. For T1-A versus T2-A, 124 and 261 of the 610 metabolites were increased (20.33%) and decreased (42.79%), respectively. For T1-A versus T2-V, 6 and 8 of the 594 metabolites were increased (1.01%) and decreased (1.35%), respectively. For T1-V versus T2-A, 125 and 256 of the 609 metabolites were increased (20.53%) and decreased (42.04%), respectively.

### 2.2. Effects of the Storage Method on Antioxidant Activity

Three measurement methods were used to evaluate the effects of the storage method on the overall antioxidant activities of mulberry leaves. FRAP assays were performed to measure the trivalent iron reduction ability. The DPPH radical scavenging capacity (DRSC) and ABTS radical scavenging capacity (ARSC) analyses were based on the single-electron transfer capacity of antioxidants to ABTS/DPPH radicals, which thus formed electron pairs.

#### 2.2.1. FRAP

The FRAP data for the mulberry leaf extracts subjected to the four storage conditions are shown in Table 1. Samples G1-T1-A, G1-T1-V, G1-T2-V, and G1-T2-A exhibited high FRAP values (35.99, 34.39, 34.23, and 27.75 mg TE/g dry sample, respectively). Samples G2-T2-A, G2-T1-V, G2-T2-V, and G2-T1-A exhibited low FRAP values (22.73, 23.49, 23.82, and 24.36 mg TE/g dry sample, respectively).

#### 2.2.2. ARSC

The ARSC values of the mulberry leaf extracts subjected to the four storage conditions are shown in Table 1. Samples G1-T2-V, G1-T2-A, G2-T1-V, G2-T2-V, and G1-T1-V exhibited high ARSC values (78.51, 76.50, 75.77, 75.52, and 75.21 mg TE/g dry sample, respectively). Samples G1-T1-A, G2-T2-A, and G2-T1-A exhibited low ARSC values (73.20, 74.94, and 72.85 mg TE/g dry sample, respectively).

#### 2.2.3. DRSC

The DRSCs of the leaf extracts subjected to the four storage conditions are shown in Table 1. Samples G1-T2-V, G1-T1-V, G1-T1-A, and G2-T2-V exhibited high DRSC values (22.24, 21.45, 20.13, and 19.13 mg TE/g dried sample, respectively). Samples G2-T1-A, G2-T1-V, G2-T2-A, and G1-T2-A exhibited low DRSC values (16.33, 18.00, 18.25, and 19.02 mg TE/g dried sample, respectively).

The experimental results showed that the antioxidant activity of the mulberry leaves was mainly influenced by the vacuum level and, secondarily, by the temperature. Vacuum storage of mulberry leaves at room temperature was the best condition for preserving antioxidant activity (T2-V > T1-V > T1-A > T2-A) in G1, and vacuum conditions also showed better preservation in G2 (T2-V ≈ T1-V > T2-A ≈ T1-A) (Table 1).

### 2.3. Comparison of the Primary Metabolites in the Growth Period of Mulberry Leaves

From the above results, we can see that both vacuum storage and refrigeration can better preserve the primary metabolites in mulberry leaves. However, the storage methods had different trends in their effects on the antioxidant activity of mulberry leaves at different growth stages. The reason for these results may be due to the changes in the types and contents of primary metabolites in mulberry leaves during the growth process.

#### 2.3.1. Qualitative and Quantitative Analyses of Primary Metabolites

**Figure 5:**
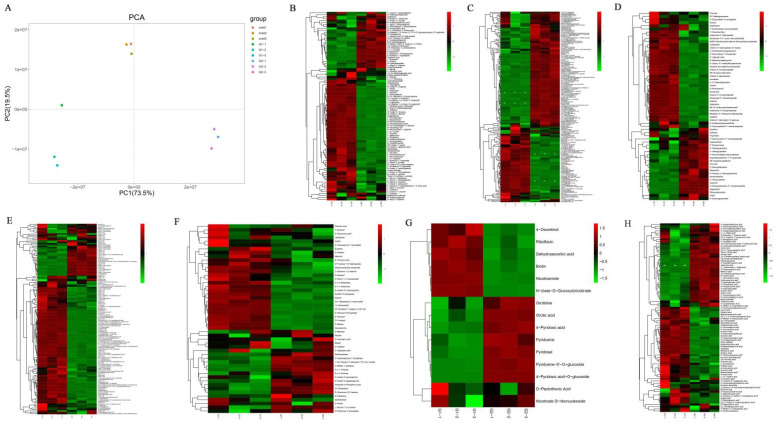
Qualitative and quantitative analyses of primary metabolites in G1 and G2. (**A**) PCA of primary metabolomic data of G1 and G2. (**B**) B–H: Heatmap of primary metabolite data of G1 and G2. (**B**) Amino acids and derivatives, (**C**) phenolic acids, (**D**) nucleotides and derivatives, (**E**) lipids, (**F**) saccharides and alcohols, (**G**) vitamins, and (**H**) organic acids. The heat maps show the relative contents of metabolites, where each line represents a metabolite. The red and green segments represent a relatively high and low abundance of metabolites, respectively. ND: not found.

UPLC-MS/MS was used to evaluate the compounds between G1 and G2. PCA, which was used to confirm the overall metabolic differences, revealed scores for the first two main components of 73.5% and 19.5%, respectively. The results showed that the G1 and G2 samples each formed a cluster (Figure 5A), indicating that the metabolites differed significantly between them. In addition, hierarchical cluster analysis was performed to identify various primary metabolite accumulation patterns in G1 and G2 (Figure 5B–H).

#### 2.3.2. Differentially Accumulated Metabolite (DAM) Identification, Functional Annotation, and KEGG Enrichment Analysis

**Figure 6:**
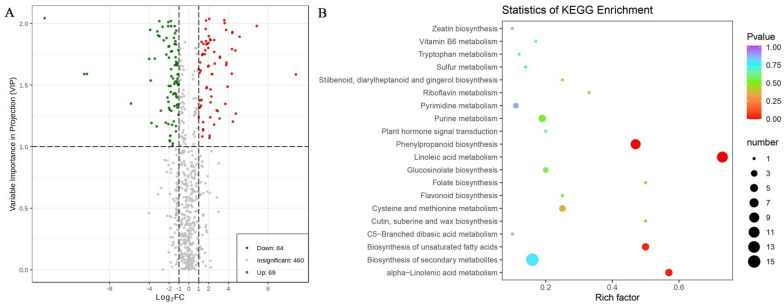
DAMs between G1 and G2. (**A**) The volcano plot shows the primary metabolites’ expression levels between G1 and G2. (**B**) Bubble diagram depicting the pathway analysis of the effects of storage on mulberry leaves’ metabolism.

In total, 153 DAMs were identified between G1 and G2. Volcano plots were generated to depict the DAMs between G1 and G2 (Figure 6A). Overall, 69 metabolites (2 amino acids and derivatives, 42 phenolic acids, 8 nucleotides and derivatives, 1 organic acid, 11 lipids, 1 saccharide and alcohol, and 4 vitamins) of 153 total metabolites (17 amino acids and derivatives, 56 phenolic acids, 10 nucleotides and derivatives, 6 organic acids, 59 lipids, 1 saccharide and alcohol, and 4 vitamins) were upregulated (45.10%); 84 metabolites (15 amino acids and derivatives, 14 phenolic acids, 2 nucleotides and derivatives, 5 organic acids, and 48 lipids) of the 153 total metabolites were downregulated (54.90%).

The KEGG database was used to annotate the functions of the DAMs in G1 and G2. The results of the metabolic pathway analysis are shown in Figure 6B. In total, 47 of the 153 primary metabolites were annotated by KEGG. The KEGG classification and enrichment results demonstrated that these 47 different metabolites were components of 20 metabolic pathways. Specifically, 15 metabolites were active in the secondary biosynthesis pathway, 11 metabolites were active in the linoleic acid metabolism pathway, and 9 metabolites were active in the phenylpropanoid biosynthesis pathway.

Note: Each bubble in the bubble diagram represents a metabolic pathway. The abscissa represents the size of the impact in the topology analysis; the larger the value, the higher the enrichment degree. The vertical coordinate and bubble color represent the negative natural log of the *p*-value of the enrichment analysis. The darker the color is, the smaller the *p*-value is and the more significant the enrichment degree is. The lighter the color is, the greater the *p*-value is and the lower the significance of the channel is.

## 3. Discussion

### 3.1. Effects of Storage Methods on Types, Contents, and Antioxidant Activities of Primary Metabolites in Mulberry Leaves

At present, mulberry leaves are mainly consumed as vegetables, tea, noodles, mulberry powder additives, and mulberry juice additives [1]. However, the content and activity of the primary metabolites in mulberry leaf must be ensured during the storage and addition processes. Many vegetables experience spoilage after harvest. Plant cells remain active after harvest and can respond to environmental stimuli [8]. Since ancient times, various food processing techniques have been used to ensure the off-season availability and freshness of vegetables [9]. Timely drying reduces the water content and allows safe storage while increasing the lipid, protein, and carbohydrate content [10,11]. It has been shown that dried fruits are not only an important source of vitamins, minerals, and fiber, but they also provide large amounts of bioactive components or phytochemicals [11]. The choice of a drying method has a great influence on the nutritive and non-nutritive compounds in fruits and vegetables [12]. The results of Hu et al. [13] showed that freeze-drying better preserved the types, contents, and antioxidant activities of the secondary metabolites of mulberry leaves. The way in which mulberry leaves are stored after processing is also an important factor in how the nutrients are better preserved. Studies have shown that factors such as temperature, moisture, carbon dioxide (CO_2_), and oxygen (O_2_) during storage affect the rate of food spoilage [12]. Appropriate storage techniques are an effective solution for vegetable spoilage. Compared to room-temperature storage, food storage at 4 °C can prevent the growth fungi and bacteria that cause spoilage [14]. Vacuum storage can significantly reduce oxygen and airborne microorganisms to slow down oxidative deterioration reactions [14] and preserve plant nutrients [15]. Therefore, we investigated the effects of different storage conditions (i.e., vacuum or non-vacuum, 4 °C or room temperature) on the nutrients in freeze-dried mulberry leaf powder during storage.

Vitamins are essential components for the metabolism of protein, carbohydrates, and fat [16]. In daily life, fresh vegetables are important sources of vitamins, including vitamin C (C_6_H_8_O_6_). In the absence of fresh vegetables, dehydrated vegetables can provide the necessary vitamins. During vegetable storage, temperature, oxygen, light, moisture, pH, storage time, enzymatic modification, and trace metal elements can have various effects on vitamins, including degradation reactions [17]. Vitamin C undergoes oxidation reactions during storage. Vitamins A (C_20_H_30_O) and E (C_29_H_50_O_2_) may also be destroyed [17]. During storage, vegetables’ primary metabolite contents exhibit specific trends. In particular, if the ascorbic acid content is comparatively stable during storage, most other primary metabolites are also preserved [18]. Although UPLC-MS/MS did not reveal the presence of ascorbic acid, dehydroascorbic acid (C_6_H_6_O_6_) was detected. Ascorbic acid and dehydroascorbic acid can be interconverted through redox reactions, and both have biological activities [19]. As antioxidants, ascorbic acid and dehydroascorbic acid help mitigate the effects of oxidative stress, thereby preventing chronic diseases (e.g., heart disease) [20]. The dehydroascorbic acid content in mulberry leaves was significantly reduced after storage in paper bags at room temperature, indicating that the environment was not conducive to vitamin preservation. During the storage of freeze-dried fresh peppers (bell pepper), Rahman et al. [21] found that the loss of ascorbic acid was proportional to the moisture content of each sample. The significant decrease in mulberry leaf dehydroascorbic acid content under the T2-A storage conditions (i.e., packed in paper bags and stored at room temperature) might be related to the absorption of moisture in the air through the paper bag, which led to increased moisture content. 

Lipid oxidation is a primary mechanism underlying the deterioration of vegetable quality. This mechanism is affected by many factors, including storage temperature and oxygen concentration. The oxidation level and type of fat/oil in vegetables may greatly affect the stability and sensory properties of primary metabolites. Differences in fat/oil types may lead to differences in vegetable quality [22]. Both low-temperature and vacuum storage can promote the preservation of mulberry leaf lipids and reduce lipid oxidation. It has been shown that vacuum packaging can strongly inhibit lipid oxidation independently of storage temperature [23]. The lipid types and contents within samples stored at room temperature under non-vacuum conditions exhibited significant changes, presumably because unsaturated lipids in these samples underwent spontaneous oxidation reactions under aerobic conditions to form peroxides [24]. Lipid oxidation can also lead to the degradation of fat-soluble vitamins and essential fatty acids, as well as the formation of toxic compounds (e.g., malondialdehyde (C_3_H_4_O_2_)) [25]. 

Phenolic acid is positively associated with free radical scavenging activity. It can inhibit lipid oxidation by scavenging free radicals and converting phenol free radicals into non-oxidative, low-energy forms [26]. Gallic acid, caffeic acid, chlorogenic acid, protocatechuic acid, and ferulic acid are the main phenolic acids in mulberry [27]. Gallic acid and its derivatives are effective antioxidants, with effects that are three times as strong as those of vitamin C or E [20]. The chromatography analysis in this study showed that the phenolic acid contents significantly decreased in mulberry leaves that were stored at room temperature under non-vacuum conditions. Additionally, the antioxidation analysis showed that mulberry leaves stored at room temperature under non-vacuum conditions had a reduced ability to scavenge free radicals, resulting in lower antioxidant activity compared with that of the low-temperature storage and vacuum storage experimental groups. 

The concentration and variation of organic acids are considered to play an important role in maintaining the nutritional value and quality of foods, allowing for changes in flavor, color, and aroma, and they are important indicators of food maturity, spoilage, and fermentation [27]. Aliphatic carboxylic acids, such as tartaric acid (C_4_H_6_O_6_), oxalic acid (C_2_H_2_O_4_), citric acid (C₆H₈O₇), and malic acid (C_21_H_28_O_3_), are common organic acids with good anti-browning effects [28]. It is possible that the mulberry leaves maintained a better morphology and color while being freeze-dried, so the contents of aliphatic carboxylic acids did not change significantly in various storage environments. The benzoic acid series and the cinnamic acid series are the main aromatic carboxylic acids [28]. In this experiment, the contents of benzoic acid series substances at room temperature in a non-vacuum showed significant differences compared with those obtained with the other three storage methods. In the mangiferous acid biosynthetic pathway, the contents of phosphoenolpyruvate and mangiferous acid (C_30_H_48_O_3_) were significantly downregulated, and the contents of their downstream amino acid products (tyrosine (C_9_H_11_NO_3_), phenylalanine (C₉HNO_2_), and tryptophan (C_11_H_12_N_2_O_2_)) were also significantly down-regulated. However, the products of the muconic acid biosynthetic pathway (3,4-dihydroxybenzoic acid (C_7_H_6_O_4_), methyl protocatechuate (C_8_H_8_O_4_), p-hydroxybenzoic acid (C₇H₆O_3_), muconic acid (C_6_H_10_O_8_), and adipic acid (C_6_H_10_O_4_)) were significantly upregulated. This may have been due to the conversion of phosphoenolpyruvic acid (C_3_H_4_KO_6_P) and 3-dehydromuconic acid into 3,4-dihydroxybenzoic acid (C_7_H_6_O_4_) in the presence of air, water, sunlight, and oxygen in a non-vacuum storage environment and the upregulation of a range of downstream compounds. Nevertheless, the principles that produce the conversion of the above compounds are not yet known.

The amino acid compositions and contents also changed substantially during storage. These changes might have been caused by altered protein structures or metabolic reactions. Storage temperature, time, and light conditions can also influence amino acid composition [24]. After mulberry leaves were subjected to storage at room temperature under non-vacuum storage conditions, the contents of various amino acids (e.g., histidine (C_6_H_9_N_3_O_2_), lysine (C_6_H_14_N_2_O_2_), arginine (C_6_H_14_N_4_O_2_), phenylalanine, and their derivatives) were significantly reduced. However, the contents of N-acetyl-L-phenylalanine (C_11_H_13_NO_3_), methyldopa (C_10_H_13_NO_4_), and lysine butyrate (C_10_H_22_N_2_O_4_) were markedly increased. These results indicated that the proteins and amino acids, among other substances, in mulberry leaves may undergo decomposition and transformation reactions during storage. Proteins are hydrophilic, which enables them to extensively interact with other compounds. The decomposition of proteins and amino acids during storage may affect mulberry leaves’ flavor and stability [24]. 

Carbohydrates are closely associated with protein, lipid, nucleic acid, and secondary biomass metabolism [24]. After mulberry leaves were subjected to storage at room temperature under non-vacuum storage conditions, the contents of sugar derivatives and alcohols were significantly reduced, indicating that the mulberry leaves’ quality declined [24]. The contents of xylitol (C_5_H_12_O_5_), ribitol (C_5_H_12_O_5_), arabitol (C_5_H_12_O_5_), glucurono-6,3-lactone (C_6_H_8_O_6_), sorbitol (C_6_H_14_O_6_), and mannitol (C_6_H_14_O_6_) significantly increased, indicating that the long-chain carbohydrates (e.g., starch) in mulberry leaves were degraded into monosaccharides. Moreover, free sugars were partially degraded into sugar derivatives during storage. These findings were similar to the results of a brown rice storage experiment [24]. In addition, the organic acid–sugar balance is directly related to vegetable flavor [20]. The malic acid content increased during exposure to storage at room temperature under non-vacuum storage conditions, indicating that the glucose in mulberry leaves was decomposed by microorganisms and converted into malic acid [29]. Moreover, the γ-aminobutyric acid content was significantly reduced, indicating that the mulberry leaves were in an aerobic state [30]. The organic acid–sugar imbalance in mulberry leaves indicated that the mulberry leaves’ flavor deteriorated with room-temperature storage in paper bags.

The experimental results showed that the type, content, and antioxidant activity of the primary metabolites of mulberry leaves were influenced by the storage methods. Vacuum packaging was the most effective storage method. A study by Rinaldi et al. [31] showed that vacuum packing at 3 °C was an effective method for storing cassava. However, another study showed that cassava produced some disruption of the parenchyma cell wall structure after refrigeration, indicating chilling injuries [14]. This may be the reason for why the antioxidant activity of mulberry leaves after low-temperature vacuum storage was lower than that after room-temperature vacuum storage. The mulberry leaves that were not stored in a vacuum at room temperature showed the worst storage effects due to the influences of air, water, carbon dioxide, and other factors.

### 3.2. Effect of the Growth Period on the Types and Contents of Primary Metabolites in Mulberry Leaves

After defining the storage conditions, it is also essential to determine the harvesting period to ensure the intake of nutrients from mulberry leaves. The results of the MMN analysis indicated no obvious differences in the types and numbers of primary metabolites throughout the mulberry tree growth process, although the differences in their expression levels were statistically significant (Figure 6B). By analyzing the primary metabolites, we revealed a total of 613 metabolites. Of these, 153 exhibited significant changes in expression levels during mulberry tree growth; 69 were significantly upregulated (mainly phenolic acids), and 84 were significantly downregulated (mainly lipids). The KEGG analysis successfully annotated 47 of these 153 metabolites into 20 signaling pathways, including those for the biosynthesis of secondary metabolites, linoleic acid metabolism, and phenylpropanoid biosynthesis. With respect to antioxidant activity, G1 had greater activity than G2 (Table 1). Changes in the expression levels of primary metabolites during mulberry tree growth may have occurred for the following reasons. First, during plant growth, nutrients and energy in the old leaves could be transferred to the developing leaves, thus enabling the use of limited nutrients for growth, reproduction, and defense [32]. Second, lipids are important components of cell membranes. They store energy, aid in growth, and support the development of mulberry trees [33]. A significant decrease in lipid expression may be caused by a lack of energy during mulberry tree growth. Third, the synthesis of phenolic acid is affected by plant growth regulators (i.e., gibberellin and abscisic acid (C_15_H_20_O_4_)) [34]. Fourth, phenolic acids (e.g., chlorogenic acid, benzoic acid (C_7_H_6_O_2_), gallic acid, ferulic acid, coumaric acid (C_6_H_4_O_4_), p-hydroxybenzoic acid, caffeic acid, and coumarin (C₉H₆O_2_)) may be related to the adaptation and defense mechanisms of plants [35,36]. 

In summary, storage methods and harvesting periods can affect the types, contents, and activities of primary metabolites in mulberry leaves. UPLC-MS/MS revealed that compared with the other three groups, the primary metabolite contents changed significantly in the mulberry leaves subjected to storage at room temperature under non-vacuum storage conditions. In contrast, the analysis of antioxidant activity showed that the activity of the primary metabolites of mulberry leaves was significantly reduced under paper bag storage conditions. These changes presumably occurred because the paper bag could not completely prevent the entry of room air, such that the mulberry leaves came into contact with oxygen and microorganisms, which resulted in oxidation or decomposition reactions that led to the deterioration of the mulberry leaves. Therefore, vacuum storage conditions are ideal for preserving mulberry leaves’ primary metabolites. Although there were no significant changes in the types of primary metabolites in mulberry leaves at different picking stages, the antioxidant activity of young mulberry leaves was significantly higher than that of old leaves. When the consumption of fresh mulberry leaves is not guaranteed, people can ensure the intake of nutrients by consuming young mulberry leaves that have been vacuum-preserved after freeze-drying.

## 4. Materials and Methods

### 4.1. Plant Material

White mulberry (*Morus alba* L.) leaves were harvested from an experimental field of South China Agricultural University (23.24° N, 113.64° E). The first harvest was performed after 3 months of growth (growing period 1, G1) after the mowing of the mulberry trees, and the plants were allowed to regrow. The second harvest was performed 6 months after the first harvest (growing period 2, G2) as a parallel group. Mulberry leaves were harvested from all mulberry plants in the experimental field, and 20 leaves were randomly harvested from each mulberry tree, pooled into a total sample, and then randomly grouped for the experiment.

### 4.2. Preparation of Dried Samples

Harvested mulberry leaves were completely freeze-dried in a freezer (Pilot 5-8S; Boyikang, Beijing, China) at −60 °C to a constant weight. After they were dried, the samples were evenly divided into 12 parts. Each part weighed 150 g and was stored under four conditions (*n* = 3 per condition) for 30 days.

T1-V: Samples were packed in polyamide bags and stored in a vacuum at 4 °C.

T1-A: Samples were packed in non-vacuum paper bags and stored at 4 °C.

T2-V: Samples were packed in polyamide bags and stored in a vacuum at room temperature (25 ± 2 °C).

T2-A: Samples were packed in non-vacuum paper bags and stored at room temperature (25 ± 2 °C).

### 4.3. Generation of the MicroTom Metabolic Network (MMN) Dataset

Samples were sent to Wuhan Metware Biotechnology Co., Ltd. (Wuhan, China) (http://www.metware.cn/) and analyzed using a primary-metabolite-targeted metabolomic method. Samples were pulverized by a mixing mill with zirconia beads (MM 400; Retsch) at 30 Hz for 1.5 min to obtain a powder. The resulting powder (100 mg each) was extracted with 1.0 mL of 70% methanol aqueous solution at 4 °C overnight and then centrifuged at 10,000× *g* for 10 min. The extracts were absorbed (CNWBOND Carbon-GCB SPE column, 250 mg, 3 mL; Anpel, Shanghai, China) and filtered (SCAA-104, 0.22-μm pore size; Anpel) before ultra-performance liquid chromatography tandem mass spectrometry (UPLC-MS/MS (UPLC, SHIMADZU Nexera X2, www.shimadzu.com.cn/; MS, Applied Biosystems 4500 Q TRAP, www.appliedbiosystems.com.cn/)) analysis [37]. Qualitative analysis was performed by using a stepwise multiple-monitoring-enhanced product ion strategy analysis. The MS2T data enabled more accurate analysis of the precursor ion (Q1) and product ion (Q3) values, as well as the retention time and fragmentation pattern, compared with injecting standards under the same conditions [37].

### 4.4. Measurement of Antioxidant Activity

The antioxidant activity of the extracts was analyzed according to the method of He et al. [38]. Each sample (0.2 g) was extracted with 10 mL of methanol for 24 h in the dark. The supernatant was then used to analyze the scavenging activity of the radicals 2,2-azinobis-3-ethylben-zothiazoline-6-sulfonic acid diammonium salt radical cation (ABTS) and 2,2-diphenyl-1-picrylhy-drazyl (DPPH), as well as ferric-reducing antioxidant power (FRAP). The absorbance was recorded at 517, 734, and 593 nm on a microplate reader (Varioskan LUX; Thermo Fisher Scientific, Waltham, MA, USA). Trolox was used to establish a standard line, and the scavenging activity and reducing power results were expressed as Trolox equivalent (mg TE/g dry sample).

### 4.5. Statistical Analysis

Each sample was analyzed by using three biological replicates, and the results were expressed as means ± standard deviations (*n* = 3). Principal component analysis (PCA) and other multivariate statistical analysis methods were used for sample comparisons. The Kyoto Encyclopedia of Genes and Genomes (KEGG) pathway database (http://www.kegg.jp/kegg/pathway.html) was used to annotate differential metabolites. The least significant difference (LSD) in a one-way analysis of variance (ANOVA) was employed to analyze the differences among the results by applying the SPSS software (version 21.0; SPSS Inc., Chicago, IL, USA) [39]. *p* < 0.05 was considered statistically significant.

## 5. Conclusions

Mulberry leaves’ primary metabolite contents and antioxidant activities were considerably affected by storage methods. Good preservation of mulberry leaves can provide better flavor and nutritional value. Among the methods investigated, vacuum storage at room temperature was optimal for the preservation of mulberry leaves’ primary metabolite contents and antioxidant activities.

## Figures and Tables

**Table 1:** Antioxidant capacity of mulberry leaves stored with four different methods in G1 and G2.

Picking Period	Storage Method	Storage Temperature	ARSC (mg TE/g Dried Sample)	DRSC (Mg TE/g Dried Sample)	FRAP (mg TE/g Dried Sample)
G1	A	T1	73.20 ± 2.80 ^b*^	20.13 ± 0.45 ^ab^	35.99 ± 2.11 ^a^
		T2	76.50 ± 2.03 ^ab^	19.02 ± 2.39 ^b^	27.75 ± 3.35 ^b^
	V	T1	75.21 ± 1.26 ^ab^	21.45 ± 1.85 ^ab^	34.49 ± 2.93 ^a^
		T2	78.51 ± 2.19 ^a^	22.24 ± 1.20 ^a^	34.23 ± 0.73 ^a^
G2	A	T1	72.85 ± 1.16	16.33 ± 2.46 ^B^	24.36 ± 0.36 ^A^
		T2	74.94 ± 1.17	18.25 ± 0.68 ^AB^	22.73 ± 0.65 ^B^
	V	T1	75.77 ± 2.97	18.00 ± 1.04 ^AB^	23.49 ± 1.37 ^AB^
		T2	75.52 ± 2.86	19.13 ± 0.31 ^A^	23.82 ± 0.23 ^AB^

*n* = 3. TE: Trolox equivalents. *: Different letters within the same column denote significant differences between groups (*p* < 0.05).

## Data Availability

The data presented in this study are available in the article here.

## Sample Availability

Samples of the compounds are not available from the authors.
